# Phytochemical Analysis and Antifungal Activity of *Aloe vera* Extract Against *Candida albicans*

**DOI:** 10.1155/sci5/4496097

**Published:** 2025-12-10

**Authors:** Hazem Sawalha, Hadeel Yousef, Rahaf Shalabi, Reem Hamamreh, Asma Kmail, Iman Qoraan

**Affiliations:** ^1^ Department of Biology & Biotechnology, Faculty of Sciences, Arab American University, Jenin, State of Palestine, aauj.edu; ^2^ Department of Microbiology & Immunology, Faculty of Medicine, Arab American University, Jenin, State of Palestine, aauj.edu

**Keywords:** antifungal activity, *Aloe vera*, *Candida albicans*, MIC, phytochemical analysis

## Abstract

Alternative medicine is pursued as a preference to conventional medicine due to growing resistance to antimicrobial medications. The primary purpose of this study is to assess the antifungal activity and phytochemical content of *Aloe vera* growing in the Palestinian region, with a particular emphasis on the bioactive potentials of this plant against *Candida albicans.* Extracts from whole leaves and gel were evaluated against *C. albicans* using MIC and broth microdilution methods. Findings revealed that the whole leaf extract demonstrated superior antifungal activity compared to the gel, with maximum efficiencies of 35.17% and 8.57%, respectively. Notably, the MIC50 values for whole leaf and gel extracts were approximately 75.42 mg/mL and 184.93 mg/mL, respectively. Phytochemical analysis exhibited considerable levels of bioactive proteins, sugars, and starch in whole leaf extracts, regardless of the extraction method, whereas gel extracts displayed lower quantities of these substances. The total phenol content was 1.278% in the entire leaf extract and 1.015% in the gel, while total flavonoid content was measured at 0.238% in the whole leaf extract compared to 0.1875% in the gel. The presence of phenols effective against *C. albicans* indicates its potential utility in alternative medicine for treating diseases caused by this fungus. Our study demonstrated that *A. vera* grown in the Palestinian region has significant levels of bioactive content, highlighting the importance of investigating distinct parts of *A. vera* for their antifungal therapeutic attributes. While many of these plants have been studied globally, a localized inquiry is necessary due to their unique qualities and potential differences within the Palestinian context. Climate, soil, and ecological conditions can influence a plant’s extract chemical composition and potency, leading to various therapeutic or pharmacological effects.

## 1. Introduction

Alternative medicine is often pursued as a preference to conventional medical treatments for a variety of health conditions, including cancer, skin disorders, and mental health issues. Supporters of alternative medicine emphasize that using medicinal plants provides the body with natural ingredients such as herbs, oils, and plant extracts to address individual health needs. It is commonly considered safer and less vulnerable to cause adverse side effects than traditional medical interventions. Furthermore, advocates dispute that alternative medicine offers an encompassing approach to health that does not depend on costly tools, engineered pharmaceuticals, or specialized procedures commonly associated with conventional medicine [1–3].

Herbal medicine refers to the use of medicinal plants to prevent or treat disease and ranges from the traditional and folk medicines of each country to the use of standard and commonly used herbal extracts [4].

Using medicinal plants in traditional remedies may suggest safety, but it does not guarantee effectiveness. Many medicinal plant formulations have been integral to healthcare practices in various cultures. They are utilized depending on spiritual or cultural beliefs rather than relying on a scientific basis [5]. Various factors can influence the composition and concentration of the bioactive ingredients in medicinal plants. Among these factors are environmental conditions, geographic variations, water salinity, extraction method, and season [6–9].

*Aloe vera* is a spiny succulent medicinal plant that encompasses approximately 420 species and flourishes in arid and semiarid environments. It has been recognized for its cosmetic and medicinal applications for a long time. The plant thrives well in sunny, dry environments, and well‐drained soil [10]. The leaf part of the plant retains high water content, which enables *A. vera* to endure prolonged periods of drought, whereas the gel is abundant in sugars, lipids, sterols, enzymes, vitamins, and various amino acids that contribute to its medical benefits [5, 11, 12]. *A. vera* is widely used in Palestinian folk medicine for various therapeutic intents. Enhancing our understanding of this plant’s ethnobotanical properties can be achieved through recording and analyzing its phytochemical composition. In addition, characterization of its antimicrobial effectiveness will generate vital, regionally customized scientific data that can guide local interventions involving new drugs derived from this plant and provide new evidence to contribute to its global understanding.

Commercially, the plant is sold in various forms, including gel, tea, juice, ointment, and other cosmetic and pharmaceutical formulations [5]. Due to its multifaceted healing properties, this spiny plant is believed to promote hair regrowth, facilitate wound healing, treat various skin conditions such as acne, lichen planus, and burns, as well as alleviate discomfort associated with burning mouth syndrome in addition to the plant’s effects in weight management, regulating sugar levels, and relieving symptoms of hepatitis and inflammatory bowel diseases.

One of the key interests of *A. vera* research has been its potential activity against fungi, specifically *Candida albicans*. The effectiveness of *A. vera* parts, specifically the leaf and the gel, against fungal infections has been discussed in various studies; however, each study has revealed variations in the plant’s efficacy based on the extraction method, phytochemical composition, and growth conditions [8, 13].

*C. albicans* is known for its opportunistic infections, skin, and mucous membranes, as well as systemic infections. It is also implicated in numerous healthcare‐associated bloodstream infections, thrush, chronic atrophic stomatitis, mucocutaneous candidiasis, and vulvovaginitis [14–16], accounting for approximately 70% of fungal infections worldwide [9, 17]. Additionally, this yeast may cause fatal infections and has a prognosis equivalent to septic shock with multiorgan failure. In Palestine, *C. albicans* is identified as a significant contributor to hospital‐acquired infections, in conjunction with the emergence of drug‐resistant strains. Locally, the yeast is isolated from patients with genitourinary, skin, gastrointestinal, blood, and respiratory tract infections [7]. The development of resistance to antifungal agents among *Candida species,* including *C. albicans,* has limited the options for treating infections caused by these pathogens [18]. As the resistant strains of this fungus continue to emerge, searching for alternative antifungal drugs from local natural sources becomes an essential inquiry [19].

The primary aim of this study is to assess the antifungal activity and phytochemical content of *A. vera* species native to the Palestinian environment, with a particular emphasis on the bioactive potentials of this plant against *Candida*. The study comprises both qualitative and quantitative analyses of the biofunctional components in the plant.

To the best of our knowledge, this is the first study in our region to investigate the antifungal activity of *A. vera* against *C. albicans* and to characterize its phytochemical composition. A growing body of research from diverse regions about this plant has been conducted globally; a localized inquiry is necessary due to its unique qualities and potential differences within the Palestinian context. There are meteorological, soil, and ecological conditions that can influence the chemical composition and potency of plant extracts, providing potential for various therapeutic or pharmacological effects. In Palestinian communities, *A. vera* has cultural and traditional significance, and it is widely cultivated in home gardens (see Figure 1).

Figure 1
*A. vera* as a home decorative and ornamental plant. (a) The plant grows indoors in a place with bright, indirect light. (b) The plant grows outdoors in home gardens.

**Figure:**
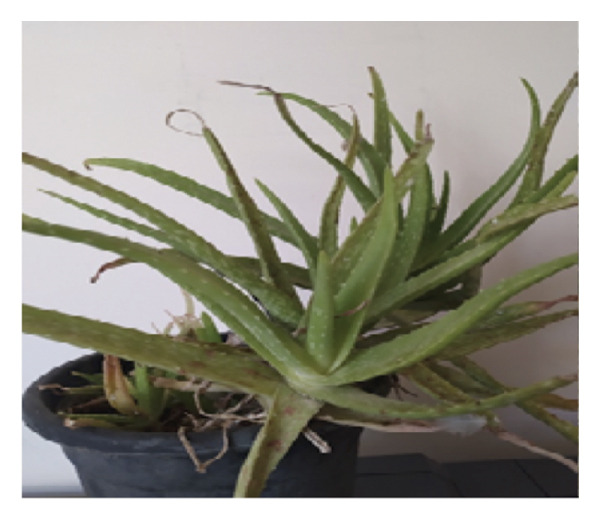
(a)

**Figure:**
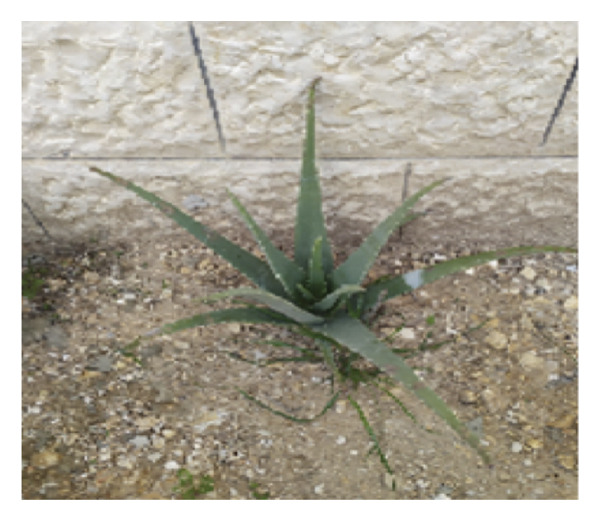
(b)

*C. albicans* was selected as the target fungus in this study due to its prevalence in our region as a common cause of opportunistic infections, its association with invasive infections among immunocompromised and hospitalized patients, and widespread occurrence of resistance to antifungal agents [20, 21]. The lack of healthcare foundations and the low health education level make *C. albicans* a center of research interest among Palestinian scientists [21–23].

## 2. Materials and Methods

**Figure 2:**
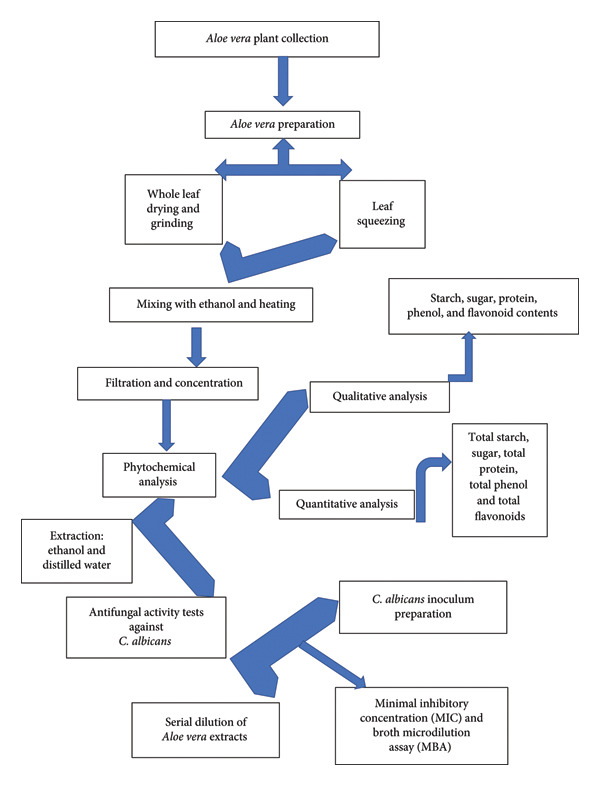
Flowchart of *Aloe vera* preparation, characterization, and testing of antifungal activity against *C. albicans*.

*A. vera* plant preparation, extraction of its bioactive components, phytochemical evaluation, and testing of the plant’s antifungal activity against *C. albicans* are summarized in Figure 2.

### 2.1. Sample Collection

Fresh leaf samples of healthy *A. vera* were collected from home gardens in the Jenin region, located at coordinates 32.48333°N 35.3°E. The plants were identified and classified according to Shtayeh and Jamous [24] (see Figure 1) and Radhakrishnan et al. [25], respectively (see Table 1). The plant identification was confirmed by the Faculty of Pharmacy staff at An‐Najah National University and given a voucher number (Pharm‐PCT‐115).

**Table 1:** Taxonomic classification of *Aloe vera.*

Rank	Taxon
Kingdom	Plantae
Phylum	Tracheophyta
Class	Magnoliopsida
Order	Asparagales
Family	Asphodelaceae
Genus	Aloe
Species	*Aloe vera*

*Note:* Including its kingdom, phylum, class, order, family, genus, and species.

### *2.2*. *A. vera* Preparation

Samples were collected from the upper leaves of one‐year‐old *A. vera* plants. After collection, the samples underwent a precise preparation process, beginning with a thorough washing using distilled water. Subsequently, the samples were divided into two 80‐g portions for further processing. The first portion was subjected to grinding, including the whole leaf and its peels, while the second portion underwent a procedure of squeezing the leaves to extract the gel. Following weighing, the plant materials were mixed with ethanol (1:2 w/v) and subjected to heating at 60°C for 60 min with continuous stirring. Both extracts were then meticulously filtered through a 0.2‐μm filter and stored in a refrigerator for 24 h [26]. Further processing involved drying the extracts at 45°C, followed by precise weighing and resuspension in sterile normal saline (0.9% NaCl) to achieve a concentration of 1000 mg/mL.

### 2.3. Preparation of Fungal Inoculum

The fungal suspension in this study was prepared from colonies cultivated on sabouraud dextrose agar for 24 h. These colonies were collected and suspended in sterile saline solution (0.9% NaCl), followed by agitation for 15 s to ensure thorough mixing. The density of the suspension was then adjusted conforming to the turbidity of a 0.5 McFarland standard, equivalent to a range of 1–5 × 10^6^ colony‐forming units per milliliter (CFU/mL) [27]. For the broth microdilution assay (MBA), conidial suspensions were prepared in RPMI‐1640 medium to achieve a final concentration of 10^3^ conidia/mL [27, 28]. The fungal isolate used in this investigation was obtained from the American Type Culture Collection (ATCC) under the serial number *C. albicans* (ATCC 90028).

### 2.4. Minimum Inhibitory Concentration (MIC) and MBA

Antifungal activity assays were performed using flat‐bottom 96‐well microtiter plates, as described by Ryan et al. [29]. Fungal cell suspensions were prepared in RPMI‐1640 medium with an optical density (OD) equivalent to 0.5 McFarland standard. Controls for growth medium only and growth medium with a fungus alone were included in each plate.

Serial dilutions of plant extracts were prepared to obtain final concentrations ranging from 100 to 12.5 mg/mL. Accordingly, 20–2.5 μL of plant extract per well was added, along with 20 μL of fungal culture and RPMI‐1640 medium, to achieve a total volume of 200 μL. After 24 h of incubation at 37°C, fungal growth was observed by visual inspection in the wells and compared with positive and negative controls. Absorbance was measured with a microplate reader at 570 nm. MIC was determined using the following formula: Percentage of inhibition = 1 − (OD test/OD positive control) × 100 [30, 31]. In addition, an MBA was performed, and the MIC 50 was calculated based on the linear equations of the standard MIC curves versus the concentration of the plant extracts [32].

### 2.5. Phytochemical Analysis

#### 2.5.1. Plant Material Preparation

Samples of *A. Vera,* including both whole leaves and gels, was collected as formerly described. The active ingredients were extracted from these samples using two distinct procedures: firstly, ethanol, and secondly, utilizing distilled water (w/v 1:2) as mentioned previously, according to Kadan et al. [26]. After extraction, the resultant extracts were dried at 45°C, weighed, and subsequently resuspended in sterile distilled water to prepare stock solutions (1 g/1 mL) for the following steps. Both extracts were subjected to chemical analysis via the procedure described below.

#### 2.5.2. Reducing Sugar Test

The total reduced sugar content in the test samples was measured using Benedict’s test. To establish positive controls, solutions of various concentrations ranging from 2% to 5% glucose were prepared in separate test tubes. Negative controls included plant sap, extraction medium, and distilled water. Subsequently, the samples in test tubes were subjected to boiling water for 5 min; thereafter, the resulting color change was monitored. The development of green, yellow, orange, red, or brick‐red color indicated the presence of sugar. With the aid of the spectrophotometer, absorbance readings were recorded at a wavelength of 570 nm, and the concentration of reducing sugar was calculated using the linear equation of the standard curve.

#### 2.5.3. Total Protein

Ninhydrin reagent (2,2‐dihydroxyindane‐1,3‐dione) was added to each sample (1:1 (v/v)). Subsequently, thorough mixing ensued, followed by immersion of samples in boiling water for 3 min. Afterward, the appearance of a distinct violet coloration was observed and measured with a spectrophotometer at 357 nm. Protein concentration was calculated according to the standard curve equation. Positive control references were established using the lysine solution spanning from 1% to 5%, as outlined by Rowe [33].

#### 2.5.4. Total Starch

Lugol’s test was performed on the samples according to Hu et al. [34]. To establish a baseline, pure starch solutions ranging from 10^−5^ to 10^−2^ were used as positive control samples and subjected to identical conditions. The coloration of the samples was measured at 357 nm using a spectrophotometer. Subsequently, the starch concentration in the samples was calculated based on the standard curve.

#### 2.5.5. Phenol and Flavonoid Content

The Folin–Ciocalteu reagent method was used to determine the total amount of phenol in the aqueous extract. Following the protocols described by Aiyegoro et al. [35] and Yadav et al. [36]. Briefly, 1 mL of plant extract was mixed with 2 mL of 2% solution of Na_2_CO_3_ and 2.5 mL of a 10% Folin–Ciocalteu reagent, and the mixture was incubated at room temperature for 15 min. Following incubation, the absorbance was measured at 765 nm using gallic acid as a standard (mg/mL). To ensure reproducibility, the experiments were repeated in duplicate. Results were expressed as gallic acid equivalents (mg/g^−1^ of extracted compound), using a standard curve for quantification.

In the assessment of total flavonoids, the aluminum chloride colorimetric screening method was applied according to Chang et al. [37]. Total flavonoid content was expressed as quercetin equivalent (mg·g^−^1 extract) based on a regression equation derived from the calibration curve.

### 2.6. Statistical Analysis

Statistical analysis of the data was done using the two‐sample tests of proportions (TSTP) to compare treatments. The results were analyzed using a level of significance when α = 0.05. The calculation was done according to the following equations [38, 39].

(1) z=P∧1−P∧2P∧1−P∧1/n1+1/n2,

two‐proportion Z‐test, pooled for $H_{0\;}:{\overset{\wedge }{P}}_{1}={\overset{\wedge }{P}}_{2}$

(2) P∧=X1+X2n1+n2.

*α*, the probability of Type I error (rejecting a null hypothesis when it is true).

*n* = sample size, *n*_1_ = Sample 1 size, and *n*_2_ = Sample 2 size.

(3) P∧=Xn=sample proportion,

where ${\overset{\wedge }{P}}_{0}=$ hypothesized population proportion, ${\overset{\wedge }{P}}_{1}=$ proportion 1, and ${\overset{\wedge }{P}}_{2}$ = proportion.

## 3. Results and Discussion

### 3.1. Antifungal Activity

**Figure 3:**
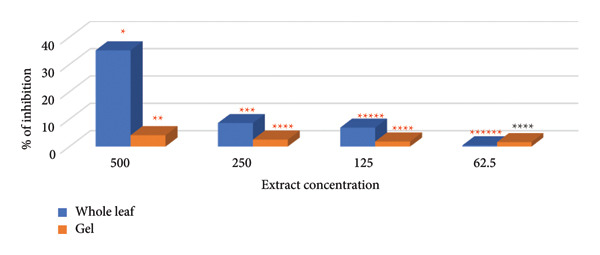
Antifungal activity of *A. vera* using MIC. “Distinct numbers of asterisks denote the significance levels across three independent experiments conducted in triplicate.”

The results of the MIC tests showed that the whole leaf and plant gel extracts displayed varying abilities to inhibit fungal growth, especially at higher concentrations. When comparing the efficacy of different plant parts used in the study, it became evident that whole‐leaf extracts were more effective against the fungus than the plant gel alone. On the other hand, the highest substantial fungal inhibition efficiency was observed at a concentration of 500 mg/mL for both extracts. The inhibition rates were 35.17% and 4.138%, respectively (see Figure 3).

### 3.2. MBA

**Figure 4:**
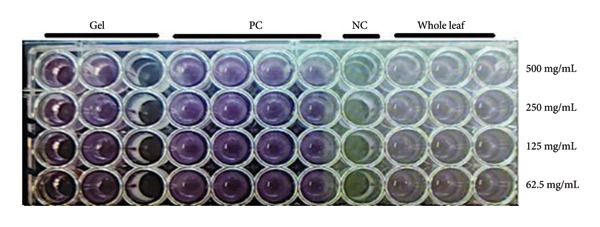
MIC results of *A. vera* extract on *C. albicans* positive control (PC)and negative control (NC).

The efficacy of the test results was validated and subsequently integrated with the MIC results to estimate the sensitivity of *C. albicans* to various plant extracts (see Figure 4).

**Figure 5:**
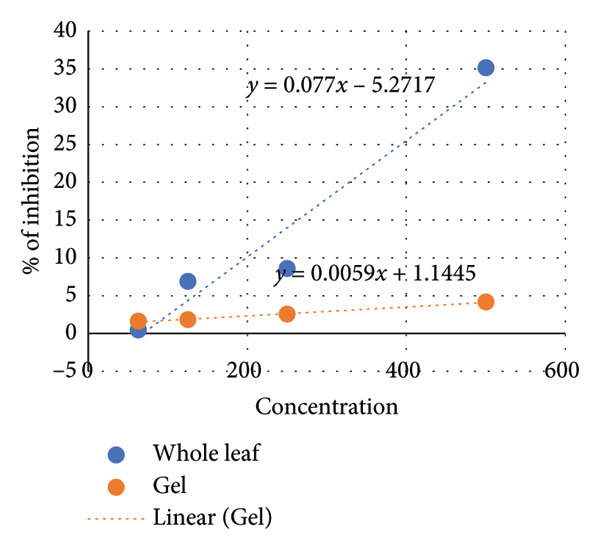
Standard curve of the microdilution method.

**Figure 6:**
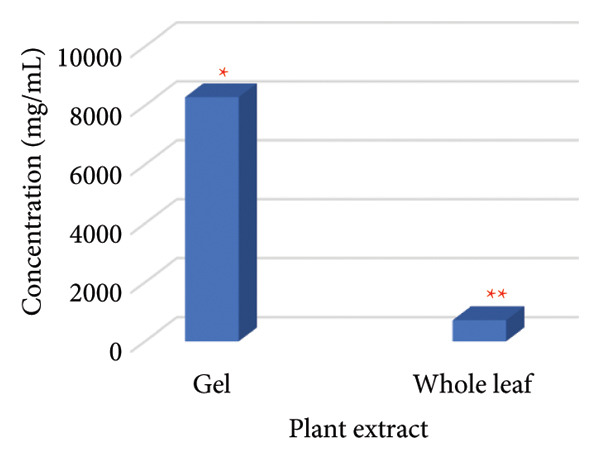
MIC50 of plant extracts against *C. albicans*. “Different numbers of asterisks denote the significant levels across three independent experiments carried out in triplicate.”

The MIC50 was determined based on the standard curve illustrated in Figure 5. The MIC values for plant extracts from both whole plant leaves and plant gel were calculated to be approximately 717.8 and 8280.6 mg/mL, respectively (see Figure 6). Since ethanol extraction yielded 7.2 and 1.2 mL of the active compound for the whole leaf extract and gel, respectively, the following equations were used to calculate the MIC 50 for the crude plant sap material, including the whole leaf extract and gel [40]:

Total active compound (mg) = MIC50 × extract volume (mL)

(4) MIC 50 crude sap=Total active compound mgCrude sap volume ml.

Crude sap volume (mL) = fresh weight (*g*)–dry weight (*g*) (assuming a density of ≈ 1 g/mL).

Thus, the MIC 50 relative to the crude sap of whole leaf and gel was calculated to be 75.42 and 184.93 mg/mL, respectively.

### 3.3. Qualitative Phytochemical Analysis

Table 2 summarizes the findings of the tested phytochemical properties of *A. vera*. The results indicate the presence of various active compounds within the tested plant samples. Phytochemical analysis revealed a consistent presence of reducing sugars, protein, and starch in all test samples, regardless of the extraction method used.

**Table 2:** Phytochemical analysis of *A. vera.*

Plant sample	Protein	Starch	Reducing sugar
Whole leaf extracted with ethanol	(+)	(+)	(+)
Gel extracted with ethanol	(+)	(+)	(+)
Whole leaf extracted with distilled water	(+)	(+)	(+)
Gel extracted with distilled water	(+)	(+)	(+)
Total	(+)	(+)	(+)

### 3.4. Quantitative Phytochemical Analysis

**Figure 7:**
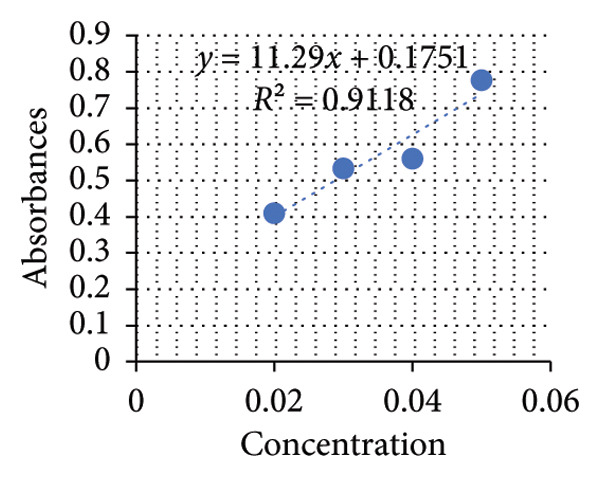
Standard curve of glucose.

**Figure 8:**
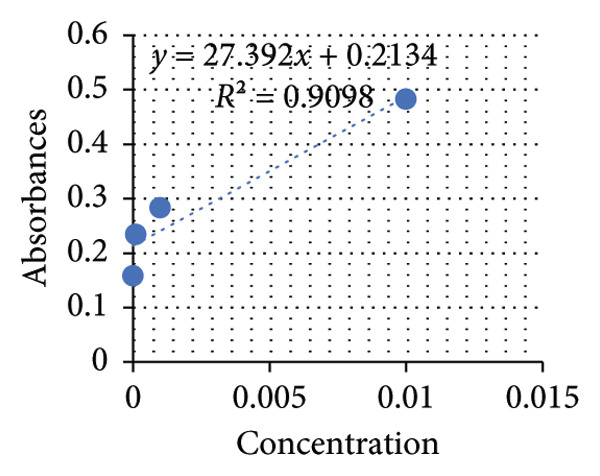
Standard curve of starch.

**Figure 9:**
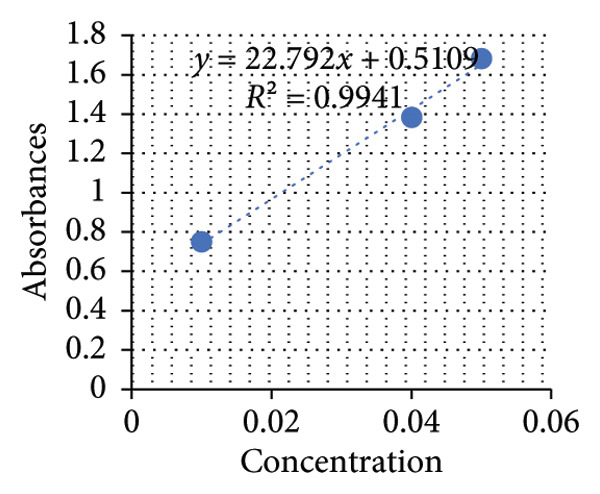
Standard curve of protein.

Based on the standard curves derived from utilizing different concentrations of positive control solutions, three distinct equations were formulated to calculate the concentrations of proteins, starch, and glucose in the plant extracts (see Figures 7, 8, 9). Applying these equations, the concentrations of these constituents in mg/mL plant extracts were determined and shown in the subsequent graphs.

**Figure 10:**
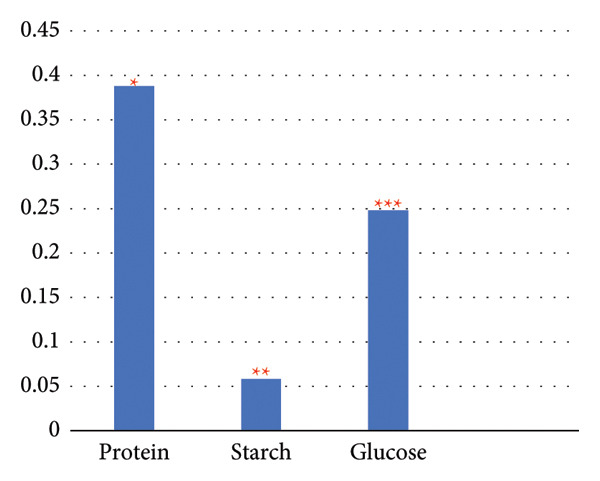
Concentration of phytochemicals of *A. vera* whole leaf extracted in distilled water. “Different numbers of asterisks denote the significant levels across three independent experiments carried out in triplicate.”

**Figure 11:**
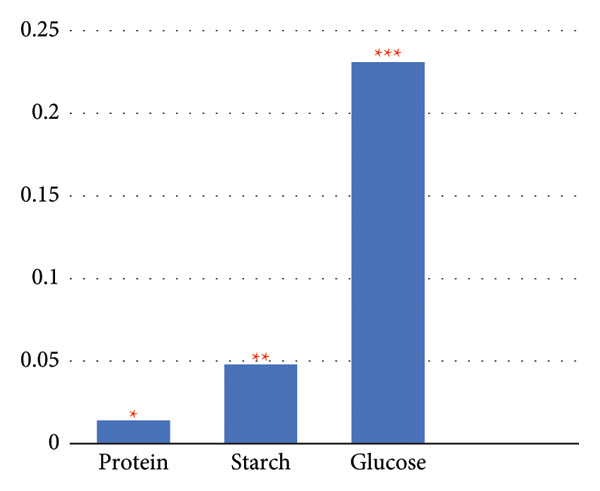
Concentration of phytochemicals of *A. vera* gel extracted in distilled water. “Different numbers of asterisks denote the significant levels across three independent experiments carried out in triplicate.”

**Figure 12:**
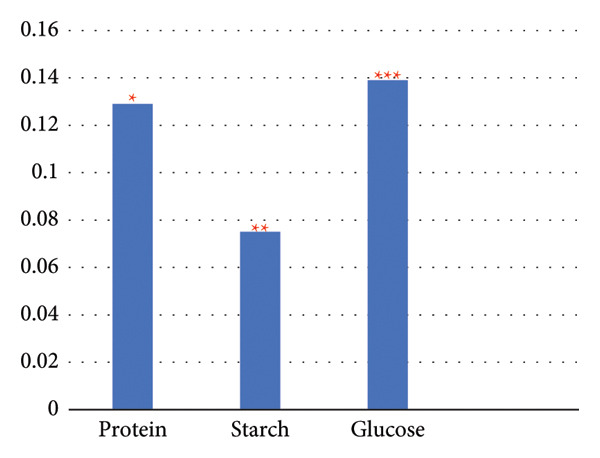
Concentration of phytochemicals. “Different numbers of asterisks denote the significant levels across three independent experiments carried out in triplicate.”

**Figure 13:**
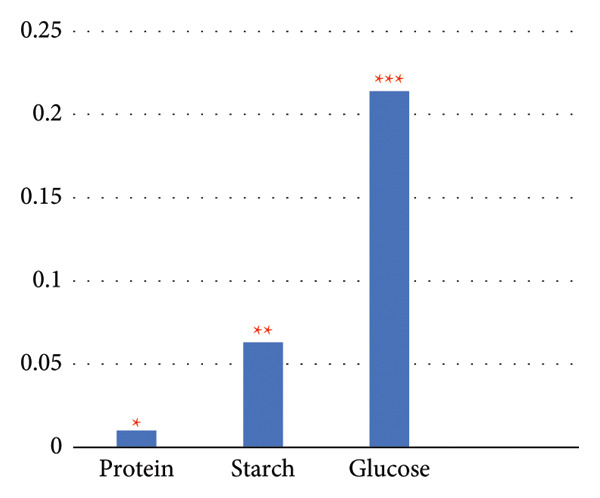
Concentration of phytochemicals of *A. vera* of *A. vera* gel extracted in EtOH whole leaf extracted in EtOH. “Different numbers of asterisks denote the significant levels across three independent experiments carried out in triplicate.”

The results unveiled significant concentrations obtained through different extraction methods. Extractions of *A. vera* leaves using either distilled water or ethanol yielded significantly higher protein and glucose levels compared to starch content. Conversely, employing the same extraction methods to obtain the juice from the same plant gave substantially higher glucose quantities, in contrast to starch and protein. In this regard, the extraction of the whole plant leaf with water yielded concentrations of 0.388 mg/mL protein, 0.058 mg/mL starch, and 0.248 mg/mL glucose (see Figure 10). However, in extraction with ethanol, these concentrations were 0.129, 0.075, and 0.139 mg/mL, respectively (see Figure 11). Similarly, in the case of gel extraction with water, the concentrations of protein, starch, and glucose were 0.014, 0.048, and 0.231 mg/mL, respectively (see Figure 12). Conversely, with ethanol extraction, these concentrations were 0.01, 0.063 mg/mL, and 0.214, respectively (see Figure 13). These findings emphasize the effect of the extraction method and the plant part on the composition of the active components in *A. vera.*

The total phenolic content was expressed as gallic acid equivalent (mg GAE/g dry extract). Analysis of methanol extracts derived from both whole *A. vera* leaf and gel revealed significant differences in their phenolic composition. The average total phenolic content of the whole leaf was estimated to be 1.278%, while the gel extract exhibited a slightly lower content at 1.015% (see Tables 3 and 4). Additionally, the total flavonoid content in these extracts was measured, giving values of 0.238% for the whole leaf extract and 0.1875% for the gel extract (see Tables 5 and 6). Plant extracts contain higher concentrations of phenols compared to flavonoids, irrespective of whether they are derived from the whole leaf or gel. On the other hand, the extract from the entire leaf demonstrates a greater abundance of phenols and flavonoids in comparison to the gel extract. These findings suggest that the whole leaf of *A. vera* serves as a richer reserve of these bioactive compounds compared to the gel part.

**Table 3:** The results of total phenol content for the whole *A. vera* leaf extracts.

Sample vol. (mL)	Replicate	Phenol content (%)	Average phenol content (%)
0.25	1	1.54	1.278 ± 0.24
0.25	2	1.49	
0.5	1	1.07	
0.5	2	1.00	
Total		5.1	

**Table 4:** The results of total phenol content for *A. vera* gel extracts.

Sample vol. (mL)	Replicate	Phenol content (%)	Average phenol content (%)
0.25	1	1.11	1.015 ± 0.028
0.25	2	0.95	
0.5	1	1.02	
0.5	2	0.98	
Total		4.06	

**Table 5:** The results of total flavonoid content for the whole *A. vera* leaf extracts.

Sample vol. (mL)	Replicate	Flavonoid content (%)	Average flavonoid content (%)
0.25	1	0.22	0.238 ± 0.04
0.25	2	0.19	
0.5	1	0.24	
0.5	2	0.30	
Total		0.95	

**Table 6:** The results of total flavonoid content for *A. vera* gel extracts.

Sample vol. (mL)	Replicate	Flavonoid content (%)	Average flavonoid content (%)
0.25	1	0.18	0.1875 ± 0.02
0.25	2	0.17	
0.5	1	0.19	
0.5	2	0.21	
Total		0.75	

Our investigation into the potential of *A. vera* in inhibiting *C. albicans* revealed compelling evidence of its antifungal properties, demonstrating effectiveness across all extracts regardless of the extraction method utilized, whether with ethanol or water. These findings emphasize the presence of active substances with potent antifungal properties.

In comparison with published studies (Table 7), Nabila and Putra [41] documented significant inhibition effects against *C. albicans* across various concentrations tested. The observed zones of inhibition, ranging from 6.25% to 50%, were determined using the disc‐diffusion method after a 72‐h incubation period. Rezvaninejad et al. [42] reported that the inhibitory concentration of *A. vera* material against the same fungus was 312.5 μL/mL, using MIC and broth dilution methods.

**Table 7:** Selected studies on *Aloe vera* extracts vs. *Candida albicans*.

Reference (author/year)	Extract type/plant part	Result	Comment
Nabila and Putra (2020) [41]	Leaf, ethanolic extract	2.5 mg/mL	Ethanol extraction concentrated antifungal compounds; strong inhibition observed.
Saniasiaya et al.(2017) [45]	Leaf, crude extract	No inhibition	Leaf extract inhibited *Aspergillus niger* but not *C. albicans*.
Cock (2008) [43]	Leaf gel components, aqueous and organic fractions	Not reported (zones of inhibition only)	Some fractions inhibited *C. albicans* in agar diffusion assay; no MIC quantified.
Rezvaninejad et al. (2022) [42]	Gel, crude gel extract	312.5 μL/mL	Inhibition of *C. albicans* at relatively low concentration; tested against multiple Candida spp.

Remarkably, our research unveiled a discrepancy in the antifungal efficacy of *A. vera* extracts depending on the plant part used in the extraction process. The whole leaf extracts, encompassing sap and peel components, exhibited superior efficacy against the fungus compared to gel extracts. The results of this study revealed that the whole leaf extract of *A. vera* grown in Palestine has inhibitory capacity against the pathogenic fungus *C. albicans*. The 50% growth inhibitory concentration (MIC50) reached approximately 75.42 mg/mL, which is considered high compared to other studies conducted on different *A. vera* cultivars in diverse geographic environments. However, what is striking in this study is that the whole leaf extract was approximately 10 times more effective than the inner gel extract, which had a MIC50 of approximately 184.93 mg/mL. This confirms that the antifungal effectiveness lies in the outer layers of *A. vera*, which are rich in active compounds such as phenols, anthraquinones, and tannins, rather than in the gel alone.

It is worth noting that this disparity in the effectiveness may not be surprising, as several studies have shown that crude *A. vera* extracts often show little effectiveness against *C. albicans*. For example, Cock et al. [43] concluded that the hydrogel extract of *A. vera* showed very weak activity, with a MIC value exceeding 400 mg/mL, and no significant inhibition of fungal growth was achieved even at higher concentrations. This suggests that the active compounds are not present at sufficient concentrations in the gel alone.

In another study by Radha and Laxmipriya [44], it was found that crude glycolic extracts of *A. vera* leaves did not show significant activity against *C. albicans.* Rather, very high concentrations were required to achieve an effect, which was attributed to the low concentration of active compounds in the crude, unpurified extract. Sansiasiya et al. [45] showed that ethanol and water leaf extracts of *A. vera* grown in a Malaysian environment exhibited no effect against *C. albicans,* while higher concentrations of the extracts, equivalent to 6.25 g/mL, were required to inhibit *Aspergillus niger* growth.

What reinforces the importance of the results of this current study is that it provides experimental evidence that Palestinian plants, despite harsh agricultural conditions and limited water resources, can exhibit antifungal properties. Aprilia et al. [46] indicated that *A. vera* plants grown in arid environments such as Palestine may exhibit lower levels of active compounds such as anthraquinones and polysaccharides, which may explain the higher MIC values compared to *A. vera* plants grown in humid or controlled environments.

Interestingly, some studies that have recorded low MIC values against *C. albicans* have used genetically enhanced *A. vera* plants or controlled cultivation conditions that result in higher concentrations of active ingredients. Hamman [47] demonstrated that processed or commercial *A. vera* varieties can achieve MICs as low as 100 mg/mL, but this is largely based on extract purification and isolation of active compounds.

Thus, what distinguishes this research is not only the discovery of an inhibitory activity in the Palestinian *A. vera* plant but also the confirmation that this activity manifests despite difficult environmental conditions, and without genetic interventions or advanced laboratory purification. This makes it a plant with significant therapeutic potential, which may be refined in the future through improved extraction methods or by focusing on isolating and testing the active compounds individually.

These collective findings underline the potential of *A. vera* as a natural antifungal agent and stress the importance of utilizing different extraction methods and plant parts in harnessing its therapeutic features against fungal infections.

The results of the phytochemical analysis revealed that *A. vera* contains a comprehensive range of compounds, including phenols, flavonoids, amino acids, proteins, vitamins, and carbohydrates, albeit in varying proportions depending on their localization within the plant and the extraction method employed. These constituents explain the efficacy of this plant’s extracts in inhibiting *C. albicans* across different levels and with various mechanisms. The leaf part of *A. vera* is a valuable source of vitamin E and moderate amounts of vitamin C and A, while containing certain amounts of proteins, giving this part its antioxidant potential. However, the gel part has the highest antioxidant activity due to the presence of vitamin A, B1, B2, B6, B12, C, E, and folic acid [48].

Furthermore, phenols and flavonoids emerge as central substances equipped with vast antifungal effects [49, 50]. The results revealed a pronounced existence of these bioactive substances within the plant matrix. Specifically, the whole leaf extract exhibited concentrations of 1.278% and 0.238% for phenols and flavonoids, respectively, while the gel extract displayed slightly lower quantities at 0.975% and 0.1875% in the same order. Consequently, the superior efficacy of whole leaf extracts against fungus was reinforced by their substantially lower inhibitory concentration (75.42 mg/mL), compared to the gel extracts (184.93 mg/mL), which displayed a comparatively weaker antifungal effect. These results align with previous findings reported by Royani et al. [49], emphasizing the presence of phenols and flavonoids within *A. vera* extracts with antimicrobial potential. Pintos et al. [51] found that the leaf extract contains high concentrations of phenolic compounds, chromones, and anthraquinones, including α‐barbaloin, aloesin, isoaloeresin D, β‐barbaloin, and oleoresins, giving the plant its fungistatic activity at variable levels against different types of molds. Añibarro‐Ortega et al. [48] showed that the leaf matrix, rich in chromones and anthrones, demonstrated enhanced antimicrobial effect against *C. albicans*, *Penicillium*, and *Aspergillus* species, surpassing the efficacy of ketoconazole antifungals.

The plant‐derived phenols exhibit antimicrobial activity through multifaceted mechanisms, including membrane disruption, enzyme inhibition, and DNA interference. They can also disrupt cell walls, inhibit protein synthesis, and act as antioxidants, reducing oxidative stress on cells. The antimicrobial properties of this plant are dependent on the type and concentration of phenols it harbors [52]. Similarly, flavonoids exhibit important antimicrobial activities, inhibiting the growth of bacteria, fungi, and viruses. Notably, quercetin, a prevalent flavonoid, has been shown to have antibacterial effects against various pathogens [53]. However, it is worth acknowledging that slight variations exist in the activity of plant extracts and their content of active constituents when compared to some global results. This variance can be attributed to the plant’s adaptation to its specific habitat, such as the Palestinian environment, resulting in unique compositions and quantities of bioactive components. These noticeable characteristics enable the plant to thrive and adapt effectively to its environment [49].

Furthermore, chemical analysis of plant extracts has revealed significant quantities of both protein and carbohydrates, which appear to play a crucial role in inhibiting fungal growth. Notably, *A. vera* stands out as rich in novel protein weighing 14 kDa, demonstrating potent antifungal properties. This purified protein, isolated from the leaf gel via ion exchange chromatography using DEAE‐cellulose and CM‐cellulose columns, exhibited inhibitory effects against fungi such as *C. parapsilosis*, *C. krusei,* and *C. albicans* [54]. This inhibitory action is likely attributed to its diverse amino acid composition, including both essential (e.g., methionine, phenylalanine, threonine, lysine, valine, isoleucine, and leucine) and nonessential amino acids (e.g., arginine, cystine aspartic acid, glutamic acid, histidine, glycine, tyrosine, proline, hydroxyproline, serine, and alanine), which may promote membrane interactions or enzyme binding critical for its inhibitory effects [55]. Moreover, the gel content of protein exhibits antifungal activity against *C. albicans* when utilized at higher concentrations, suggesting its potential as a promising adjunct to conventional antifungal agents [56]. Proteins can exhibit antimicrobial activities through diverse mechanisms, including direct interaction with microbial cells or interference with fundamental microbial processes. Siritapetawee et al. [57] revealed that *A. vera* gel contains AVPI‐12, a protein with a molecular weight of 11,804 Da. It functions as an antiprotease by inhibiting plasmin, papain, and trypsin, thereby protecting structural proteins from enzymatic degradation. This defensive action on host tissues may also contribute indirectly to antifungal activity by targeting fungal proteases. Cabello‐Ruiz et al. [58] found that *A. vera* gel extract contains peptides with sequence homology to plant antimicrobial peptides such as defensins, indicating that these compounds may exhibit antifungal activity by compromising cell membranes or disrupting the fungal cell wall integrity.

On the other hand, studies revealed that some carbohydrate‐based compounds exhibit antimicrobial properties by interfering with microbial cell wall structures or inhibiting the growth of pathogens [59, 60]. Aloin, a glycoside of aloe‐emodin in the matrix part of *A. vera,* demonstrates antifungal properties by altering the β‐1,3‐glucan component of hyphae in *C. albicans,* specifically resistant strains, thereby diminishing its virulence [60]. Matie et al. [61] and Sahu et al. [62] revealed that the acemannan component of the plant, located in the gel part of the leaves, exhibits immune‐modulating and antiseptic properties by enhancing the immune system activities through increased proliferation of macrophages, T lymphocytes, and dendritic cells. This acylated mannose also stimulates antigen presentation, induces the release of nitric oxide molecules, and increases the formation of reactive oxygen radicals. Quezada et al. [63] found that polysaccharides, such as glucomannan and fructans, exert their antimicrobial activity by supporting gut flora through a prebiotic effect, suppressing the growth of pathogenic microorganisms such as *Clostridium perfringens,* while also acting synergistically with acemannan to suppress microbial growth and biofilm formation.

Our results emphasized that protein and carbohydrate extraction methods employing distilled water are more effective in inhibiting fungal growth compared to those utilizing ethanol. This variance in efficacy is likely attributed to the enhanced solubility of fungal inhibitors in water relative to ethanol, underscoring the importance of solvent choice in maximizing the bioactive potential of plant extract.

## 4. Conclusions

Our study demonstrated that the phytochemical content varies depending on the part of the plant utilized, highlighting the effect of geographical variations on the plants’ phytochemical composition and concentration. Higher flavonoid levels derived from *A. vera* grown in the Palestinian regions signify this outcome.

By demonstrating that crude *A. vera* preparations exhibit measurable antifungal activity against *C. albicans*, albeit less potent than concentrated extracts, our results underscore the potential of *Aloe*‐based formulations as complementary antifungal agents. Importantly, this study contributes to the existing body of knowledge by showing that the phytochemical composition and antifungal activity of *A. vera* differ significantly between the leaf and gel extracts, with distinct MIC values. This localized evidence from Palestinian‐grown *A. vera* highlights the influence of regional environmental and climatic factors on bioactivity, supporting the need for context‐specific evaluations.

Our findings also provide a cornerstone for future studies aimed at developing eco‐friendly, plant‐based antifungal therapies. Exploring synergistic interactions with conventional antifungal drugs, optimizing extraction methods, and concentrating active compounds may further enhance efficacy, particularly against resistant fungal strains. In clinical and pharmaceutical contexts, such insights offer a basis for innovative topical applications or preventive care in oral and mucosal candidiasis. Finally, future research should examine how cultivation conditions, processing efficiency, and advanced phytochemical characterization techniques may optimize antifungal potential and enable sustainable large‐scale utilization of this valuable plant.

## Ethics Statement

Samples were collected following international regulations and university guidelines. Appropriate permissions were obtained in compliance with local laws.

## Disclosure

All authors read and approved the final manuscript.

## Conflicts of Interest

The authors declare no conflicts of interest.

## Author Contributions

All authors contributed to the study concept and design. The first draft of the manuscript was written by Hazem Sawalha. The manuscript editing and reviewing were done by Iman Qoraan. Laboratory preparation and results follow‐up were conducted by Hazem Sawalha, Hadeel Yousef, Rahaf Shalabi, Reem Hamamreh, and Asma Kmail. All authors commented on previous versions of the manuscript.

## Funding

No funding was received for this research.

## Data Availability

The data that support the findings of this study are available on request from the corresponding author. The data are not publicly available due to privacy or ethical restrictions.
